# Light scattering effect of polyvinyl-alcohol/titanium dioxide nanofibers in the dye-sensitized solar cell

**DOI:** 10.1038/s41598-019-50292-z

**Published:** 2019-10-18

**Authors:** Muhammad Norhaffis Mustafa, Suhaidi Shafie, Mohd Haniff Wahid, Yusran Sulaiman

**Affiliations:** 1 Department of Chemistry, Faculty of Science, Universiti Putra Malaysia, 43400 Serdang, Selangor Malaysia; 2 Department of Electrical and Electronics Engineering, Faculty of Engineering, Universiti Putra Malaysia, 43400 UPM Serdang, Selangor Malaysia; 3 Functional Devices Laboratory, Institute of Advanced Technology, Universiti Putra Malaysia, 43400 UPM Serdang, Selangor Malaysia

**Keywords:** Chemistry, Energy science and technology

## Abstract

In the present work, polyvinyl-alcohol/titanium dioxide (PVA/TiO_2_) nanofibers are utilized as a light scattering layer (LSL) on top of the TiO_2_ nanoparticles photoanode. The TiO_2_ nanoparticles decorated PVA/TiO_2_ nanofibers display a power conversion efficiency (PCE) of 4.06%, which is 33% higher than TiO_2_ nanoparticles without LSL, demonstrating the incorporation of PVA/TiO_2_ nanofibers as LSL reduces the radiation loss and increases the excitation of the electron that leads to high PCE. The incorporation of PVA/TiO_2_ nanofibers as LSL also increases the electron life time and charge collection efficiency in comparison to the TiO_2_ nanoparticles without LSL.

## Introduction

**Figure 1:**
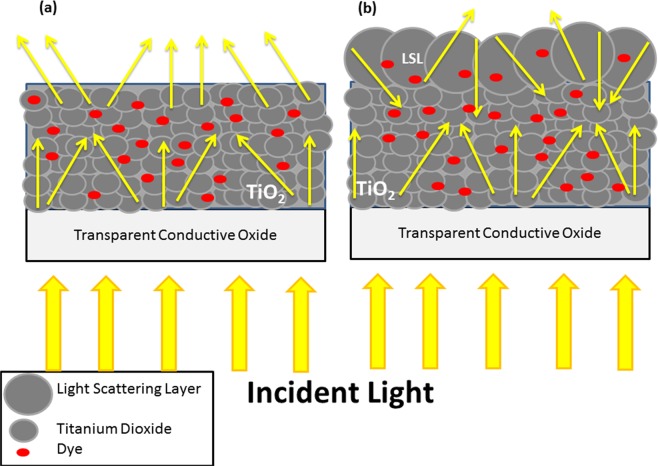
Illustration of light loss of (**a**) photoanode without light scattering layer and (**b**) photoanode with light scattering layer.

In dye-sensitized solar cells (DSSCs), light scattering layer (LSL) is important to prevent or reduce the amount of light loss during the DSSC process that could lead to reducing in power conversion efficiency (PCE). In the DSSC process, the higher the amount of light trapped on the sensitized photoanode, the more dye molecules will be excited and more voltage and current will be produced, resulting in an increase of PCE of the DSSC device^1^. Usami^2^ reported a theoretical concept of light scattering layer by introducing larger TiO_2_ nanoparticles on top of the TiO_2_ photoanode to increase the light scattering and to improve the optical absorption of the photoanodes. Figure 1 shows the illustration of light loss of the photoanode with and without LSL.

A lot of one-dimensional TiO_2_ morphological structures have been studied as LSL in DSSC such as nanofibers^3–5^, nanorods^6^ and nanotubes^7^ because they can provide a straight pathway for the electron transfer and improve the electron transport rate to reduce the recombination effect significantly^8–10^. Among those one-dimensional structures, TiO_2_ nanofibers are one of the most attractive one-dimensional nanostructure materials for LSL in DSSC because of their unique properties such as high surface area-to-volume ratio^11^ and porous structure^12,13^. Furthermore, nanofibers can be synthesized from various materials, such as natural polymers^14^, synthetic polymers^15^, carbon-based nanomaterials^16,17^, composite materials^18,19^ and semiconducting materials^20^. Zheng and Zhu^21^ reported that LSL made of gold doped TiO_2_ nanofibers via electrospinning capable of producing a PCE of 5.08% due to an increase in the light harvesting efficiency. Furthermore, cadmium doped TiO_2_ nanofibers as LSL in DSSC were successfully prepared by Motlak, *et al*.^22^ via electrospinning. A PCE of 2.95% was achieved which is higher than bare photoanode (1.54%) due to the increase in the density of the electrons and increase in the injected electrons in the photoanode with LSL that leads to increase electron lifespan and preventing electron-holes recombination. A mixed phase of copper oxide nanoparticles (CuO-Cu_2_O) prepared by microwave heating technique was used as LSL in DSSC and a higher PCE of 2.31% was achieved compared with pure DSSC without LSL (1.76%)^23^. This is due to the increase in dye loading capacity and improve light scattering ability^23^.

Herein, we introduce a facile electrospinning to prepare the PVA/TiO_2_ nanofibers as LSL. The TiO_2_ nanoparticles with PVA/TiO_2_ nanofibers as LSL exhibited a higher dye loading capacity and a better DSSC performance compared with TiO_2_ nanoparticles without LSL. Furthermore, the TiO_2_ nanoparticles with PVA/TiO_2_ nanofibers as LSL displayed a longer electron life time and higher charge collection efficiency compared with TiO_2_ nanoparticles without LSL.

## Experimental

### Materials

Ruthenizer 535-bis TBA (N719) and Iodolyte Z-100 were purchased from Solaronix SA while, 3,4-ethylenedioxythiophene (EDOT), titanium tetraisopropoxide (TTIP), polyvinyl alcohol (PVA) and titanium dioxide (TiO_2_, Degussa P25) were purchased from Sigma Aldrich. Tert-butanol (C_4_H_10_O), ethanol (CH_3_CH_2_OH), sodium hydroxide (NaOH), nanocrystalline cellulose (NCC) and acetonitrile were obtained from Merck, J. Kollin Chemicals, University of Maine and Friendemann Schmidt, respectively. Indium tin oxide (ITO 7Ω/sq) was purchased from Xinyan Technology Ltd. Deionized (DI) water (Mili-Q 18.2 MΩ.cm) was used throughout the experiments.

### Preparation of photoanodes

The TiO_2_ photoanodes were prepared using a doctor blade technique. Briefly, the TiO_2_ pastes were prepared by mixing 8 mL of ethanol with 2 g of TiO_2_ (Degussa P25). The mixture was stirred and 0.16 mL of TTIP was added into the mixture followed by sonication in an ultrasonic bath for 30 minutes. A TiO_2_ compact layer was deposited on the ITO as previously reported^24^. The doctor blade technique was then applied to deposit the TiO_2_ paste on the ITO/compact layer. The ITO/compact layer/TiO_2_ photoanodes were annealed at 200 °C for two hours using a hot plate. The photoanodes were then immersed in a dye bath solution containing 0.2 mM (N719) in the same ratio of acetonitrile and tert-butanol for 24 hours to produce sensitized photoanodes.

### Preparation of PVA/TiO_2_ nanofibers as a light scattering layer

The light scattering layer was prepared using electrospinning. 10 wt.% of PVA was dissolved in DI water and stirred at 80 °C until a clear PVA solution was formed. 0.06 M TTIP was then added into the PVA solution and stirred for 2 hours to form the electrospun solution. The as-prepared electrospun solution was transferred into a 5 mL syringe with a blunt needle. The electrospinning was performed by applying a voltage of 15 kV and the flow rate of 1.2 mL/h. The distance between the tip of the needle and the current collector was fixed to 15 cm. The current collector was the as-prepared ITO/compact layer/TiO_2_ photoanodes. The electrospun time to produce PVA/TiO_2_ nanofibers was fixed to 9.88 min^25^.

### Preparation of counter electrode

The counter electrode for the DSSC was fabricated using a previously reported chronoamperometry technique^26^. Briefly, the PEDOT/NCC counter electrode was electrodeposited on the ITO using three electrode systems where the applied voltage and deposition time used were 1.2 V and 100 s, respectively. The electrodeposited solution consisted of 1 mg/mL NCC and 10 mM EDOT. The working, counter and reference electrode used were ITO, platinum wire and silver/silver chloride, respectively.

### Device fabrication

A complete DSSC device was assembled by sandwiching both the sensitized photoanodes (ITO/compact layer/TiO_2_/LSL) and counter electrode (PEDOT/NCC). The electrolyte (Iodolyte Z-100) was injected in between the photoanodes and counter electrode. A black mask with an active area of 0.25 cm^2^ was used to analyze the DSSC performance.

### Characterization

The morphological studied was performed using the field emission scanning electron microscopy (FESEM, JEOL JSM-7600F). The crystallographic analysis of the photoanodes was performed using Shimadzu X-ray diffraction (XRD) Diffractometer with Cu Kα radiation (λ = 1.54 Å). The electrochemical impedance spectroscopy (EIS) was carried out using Autolab PGSTAT204 equipped with NOVA software. The EIS was carried out in a dark condition at open circuit potential (OCP) of 0.8 V and a frequency range between 100 kHz to 1 Hz in the presence of Iodolyte Z-100. The dye loading capacity analysis of the photoanodes was performed using Autolab Spectrophotometer UB in the range of 200–550 nm. The sensitized photoanodes were immersed into 0.1 M NaOH solution to desorb the dye (N719) molecules from the photoanodes and the solutions were tested with ultraviolet-visible (UV-Vis) analysis to calculate the amount of the dye (N719) molecules absorbed by the photoanodes. The photovoltaic analysis of the complete DSSC devices was performed using Oriel LCS-100 solar stimulator (1.5 A.M, 100 mW/cm^2^ and 100 watt Xenon lamp) equipped with a potentiostat (Autolab PGSTAT204).

## Results and Discussion

### Morphological studies

**Figure 2:**
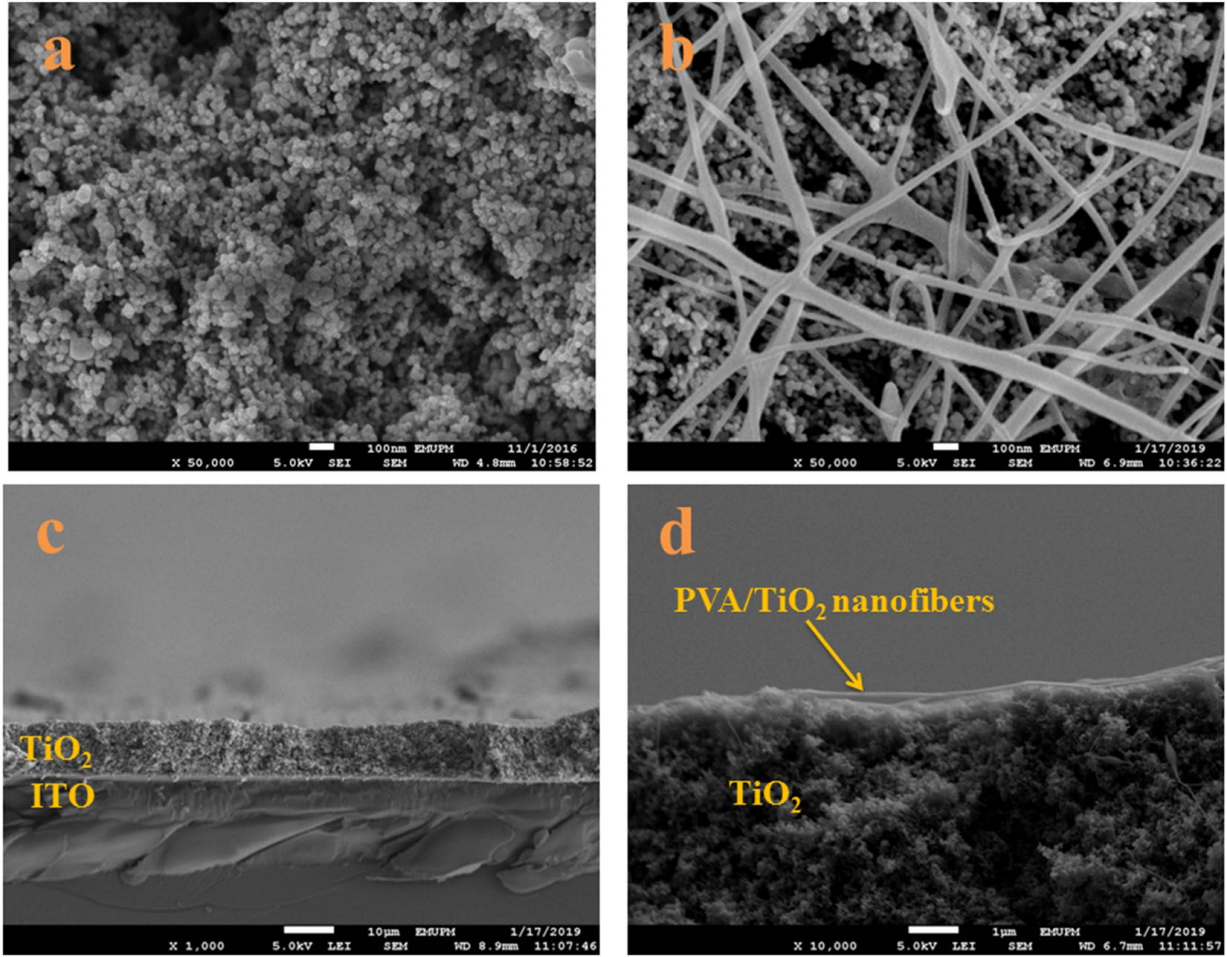
FESEM image of (**a**) TiO_2_ nanoparticles, (**b**) TiO_2_ nanoparticles with PVA/TiO_2_ nanofibers, (**c**) cross-sectional of TiO_2_ nanoparticle (**d**) cross-sectional of TiO_2_ nanoparticles with PVA/TiO_2_ nanofibers.

FESEM was performed to study the morphology of the photoanode. Figure 2 shows the FESEM images of TiO_2_ nanoparticles and TiO_2_ nanoparticles with the PVA/TiO_2_ nanofibers. Figure 2a depicts the spherical nanoparticles structure of TiO_2_ which is important in the absorption of the dye. As shown in Fig. 2b, the TiO_2_ nanoparticles with PVA/TiO_2_ nanofibers display a network of fibers that cover the surface of spherical nanoparticles of TiO_2_ with an average diameter of 45 ± 20 nm. The large diameter of PVA/TiO_2_ nanofibers than TiO_2_ spherical nanoparticles is important to create a light scattering effect to trap the sunlight and increase the PCE of the DSSC^27^. The cross-sectional of TiO_2_ nanoparticles and TiO_2_ nanoparticles with PVA/TiO_2_ nanofibers are shown in Fig. 2c,d, respectively. The results illustrate that the PVA/TiO_2_ nanofibers is thinner than TiO_2_ nanoparticles and only cover the top of TiO_2_ nanoparticles. A thin LSL is important to avoid the increase in the internal resistance that will reduce the DSSC performance^28^.

### X-ray diffraction analysis

**Figure 3:**
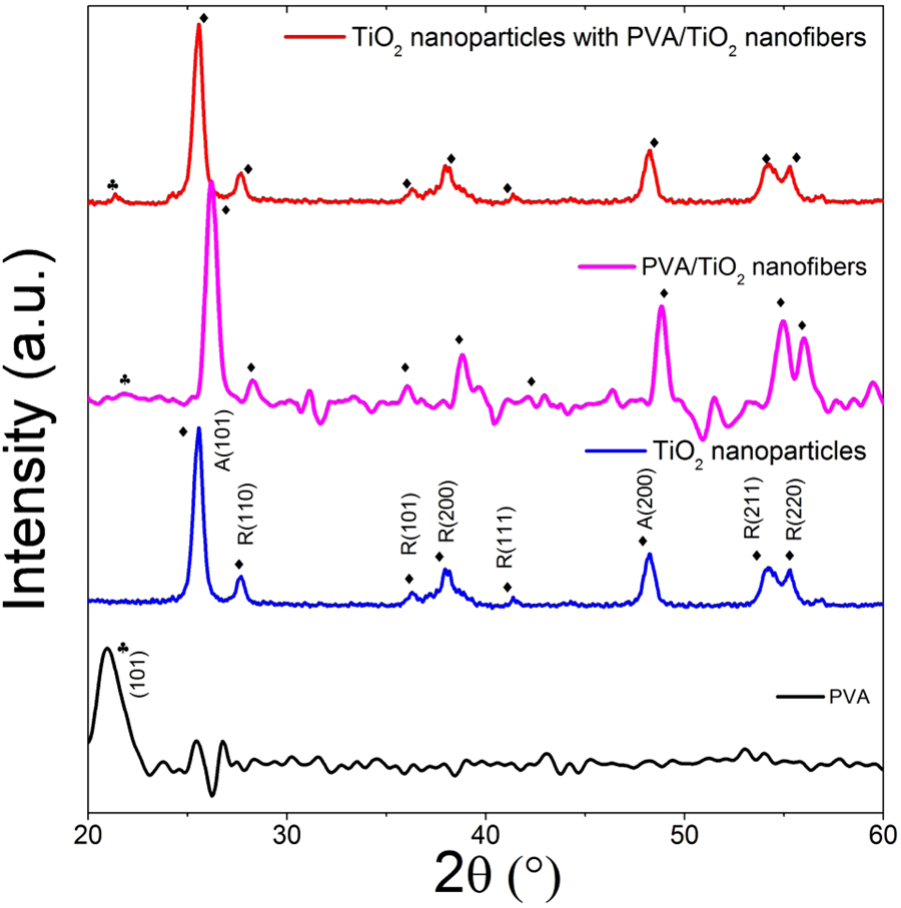
XRD pattern of TiO_2_ nanoparticles, TiO_2_ nanoparticles with PVA/TiO_2_ nanofibers and PVA/TiO_2_ nanofibers. Peaks labeled with “♦” and “♣” belong to the peaks of TiO_2_ and PVA, respectively.

XRD was performed to study the crystalline structure of the photoanodes. Figure 3 shows the XRD patterns of PVA, TiO_2_ nanoparticles, TiO_2_ nanoparticles with PVA/TiO_2_ nanofibers and PVA/TiO_2_ nanofibers. All the diffraction peaks of TiO_2_ nanoparticles can be well indexed to the anatase and rutile phase of TiO_2_ (JCPDS 01-073-1764) and the same XRD patterns were reported by Zhao, *et al*.^29^. The diffraction peak of PVA appears at around 2θ = 22° (101)^30^. PVA/TiO_2_ nanofibers displays all the peak belongs to PVA and TiO_2_. Upon addition of PVA/TiO_2_ nanofibers, one additional characteristic peak at around 2θ = 22° (101) is observed, indicating the presence of PVA^30^.

### Electrochemical impedance spectroscopy

**Figure 4:**
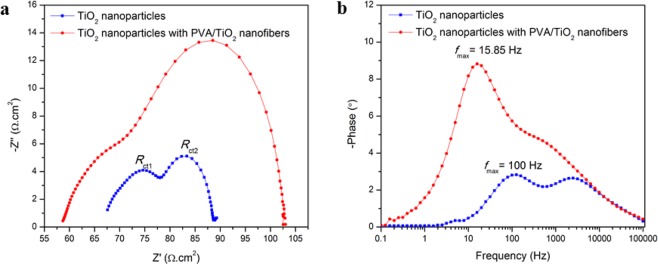
(**a**) Nyquist plot and (**b**) bode plot of TiO_2_ nanoparticles and TiO_2_ nanoparticles with PVA/TiO_2_ nanofibers.

Figure 4a displays a comparison of Nyquist plots between TiO_2_ nanoparticles and TiO_2_ nanoparticles with PVA/TiO_2_ nanofibers. Two semicircles are clearly observed: the high frequency semicircle (*R*_ct1_) corresponds to the charge transfer resistance at the counter electrode interface, while the low frequency semicircle (*R*_ct2_) is attributed to the charge transfer resistance at TiO_2_/dye/electrolyte interface^31^. Upon addition of PVA/TiO_2_ nanofibers as LSL on top of TiO_2_ nanoparticles, the *R*_ct1_ increases (Table 1) due to the presence of a new layer on top of the photoanode obstructs the movement of the electron to complete the electron-regeneration process^32^. Furthermore, TiO_2_ nanoparticles with PVA/TiO_2_ nanofibers displays a higher *R*_ct2_ (34.03 Ω.cm^2^) compared to the *R*_ct2_ of TiO_2_ nanoparticles (12.70 Ω.cm^2^) (Table 1). This is because the addition of PVA/TiO_2_ nanofibers as LSL on top of the TiO_2_ nanoparticles will increase the resistance at the TiO_2_/dye/electrolyte interface^28,33^. However, TiO_2_ nanoparticles with PVA/TiO_2_ nanofibers depicts a lower series resistance (*R*_s_) of 58.34 Ω.cm^2^ compared with TiO_2_ nanoparticles (67.60 Ω.cm^2^), demonstrating the incorporation of PVA/TiO_2_ nanofibers as LSL slightly increases the conductivity of the photoanode. This fact is supported by the conductive TiO_2_ was well blended with PVA nanofibers during electrospinning. Apart from charge transfer resistance, the charge collection efficiency (η_c_) also can be calculated from EIS analysis using formula 1^34^. TiO_2_ nanoparticles with PVA/TiO_2_ nanofibers displays a higher η_c_ (38.84%) compared with TiO_2_ nanoparticles (15.8%), indicating that incorporation of PVA/TiO_2_ nanofibers as LSL improves the light scattering effect of the photoanodes that lead to increase in η_c_.

1 $${\eta }_{c}={(1+\frac{{R}_{s}}{{R}_{ct2}})}^{-1}$$

**Table 1:** EIS parameter of the DSSCs based on TiO_2_ nanoparticles and TiO_2_ nanoparticles with PVA/TiO_2_ nanofibers.

Photoanodes	*R*_s_ (Ω.cm^2^)	*R*_ct1_ (Ω.cm^2^)	*R*_ct2_ (Ω.cm^2^)	*f*_max_ (Hz)	τ_n_ (ms)	η_c_ (%)
TiO_2_ nanoparticles	67.60	17.00	12.70	100.0	1.59	15.8
TiO_2_ nanoparticles with PVA/TiO_2_ nanofibers	58.34	29.25	34.03	15.85	10.04	36.84

As shown in Fig. 4b, Bode plot analysis was extracted from EIS analysis to study the electron life time (τ_n_) between TiO_2_ nanoparticles and TiO_2_ nanoparticles with PVA/TiO_2_ nanofibers. The *f*_max_ value of TiO_2_ nanoparticles and TiO_2_ nanoparticles with PVA/TiO_2_ nanofibers are 100 and 15.85 Hz while the τ_n_ values of TiO_2_ nanoparticles and TiO_2_ nanoparticles with PVA/TiO_2_ nanofibers are 1.59 and 10.04 ms, respectively (Table 1). The τ_n_ values of TiO_2_ nanoparticles and TiO_2_ nanoparticles with PVA/TiO_2_ nanofibers were calculated using formula 2^34^.

2 $${\tau }_{n}=\frac{1}{2\pi {f}_{max}}$$

**Figure 5:**
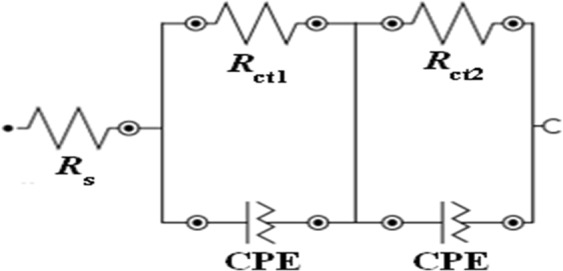
Equivalent circuit for EIS.

Upon incorporation of PVA/TiO_2_ nanofibers as LSL, the maximum frequency (*f*_max_) is shifted from a higher frequency region to lower frequency region, producing a longer τ_n_ and improves the light scattering capability of the photoanode. Figure 5 shows the electrical equivalent circuit that was constructed by fitting the EIS data. The fitting circuit models consist of *R*_s_, *R*_ct1_, *R*_ct2_ and constant phase element (CPE). The CPE indicates the inhomogeneity of the photoanode after being modified^35^. The low chi-square achieves by TiO_2_ nanoparticles (0.00109) and TiO_2_ nanoparticles with PVA/TiO_2_ nanofibers (0.00011) indicate the better suitability of the equivalent circuit with the Nyquist plot.

### Ultraviolet-visible analysis

**Figure 6:**
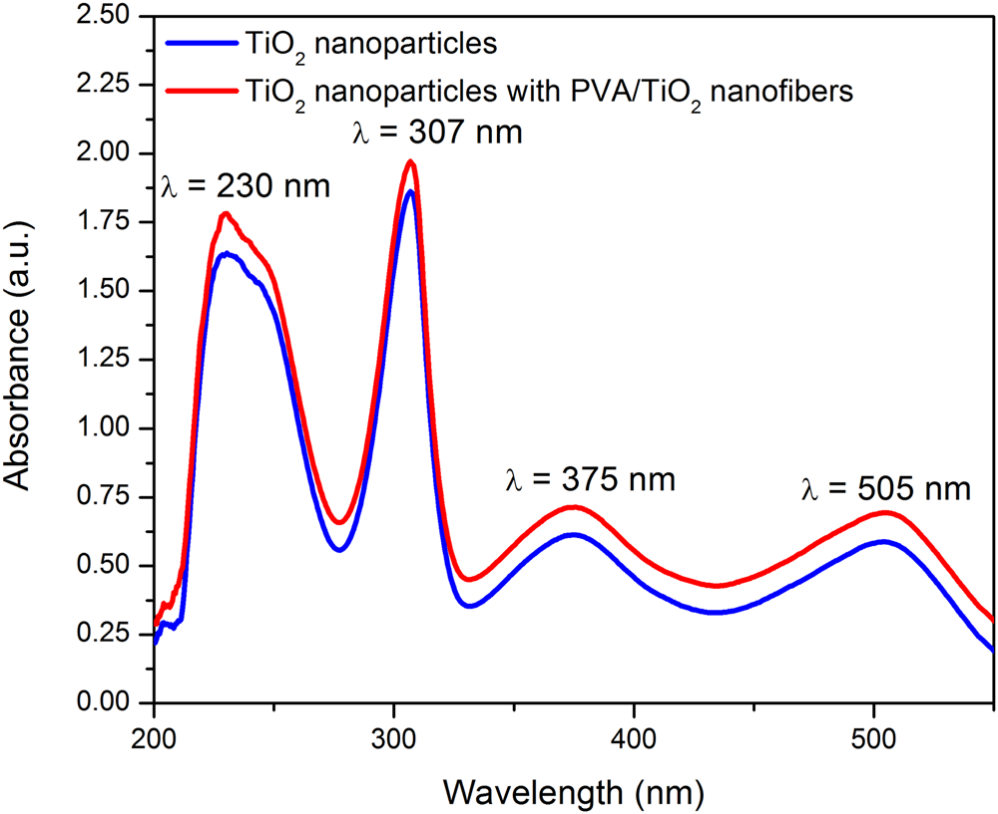
UV-Vis absorption of TiO_2_ nanoparticles and TiO_2_ nanoparticles with PVA/TiO_2_ nanofibers.

The UV-Vis analysis was performed to study the dye loading capacity of photoanodes with and without LSL. Figure 6 displays the UV-Vis absorption spectra of TiO_2_ nanoparticles and TiO_2_ nanoparticles with PVA/TiO_2_ nanofibers. Both UV-Vis spectra depict four main absorption peaks at wavelength 230, 307, 375 and 505 nm. The absorption peaks at lower energies (375 and 505 nm) are attributed to the metal-to-ligand charge transfer (MLCT) transition (4d − π*) while the absorption peaks at higher energies (230 and 307 nm) are ascribed to the ligand-centered charge transfer (LCCT) transitions (π − π*)^36^. TiO_2_ nanoparticles with PVA/TiO_2_ nanofibers displays higher absorption peaks compared to the TiO_2_ nanoparticles, indicating that the incorporation of PVA/TiO_2_ nanofibers as LSL increases the dye loading capacity of the photoanodes. The amounts of dye molecules absorbed by TiO_2_ nanoparticles and TiO_2_ nanoparticles with PVA/TiO_2_ nanofibers are 0.035 and 0.037 mmol cm^−2^, respectively. The increment in the concentration of dye molecules absorbed by the photoanode with LSL is due to the morphological structure of PVA/TiO_2_ nanofibers that increase the surface area of the photoanode, resulting to more dye molecules can be absorbed by the photoanode.

### Photovoltaic performance of the DSSCs

**Figure 7:**
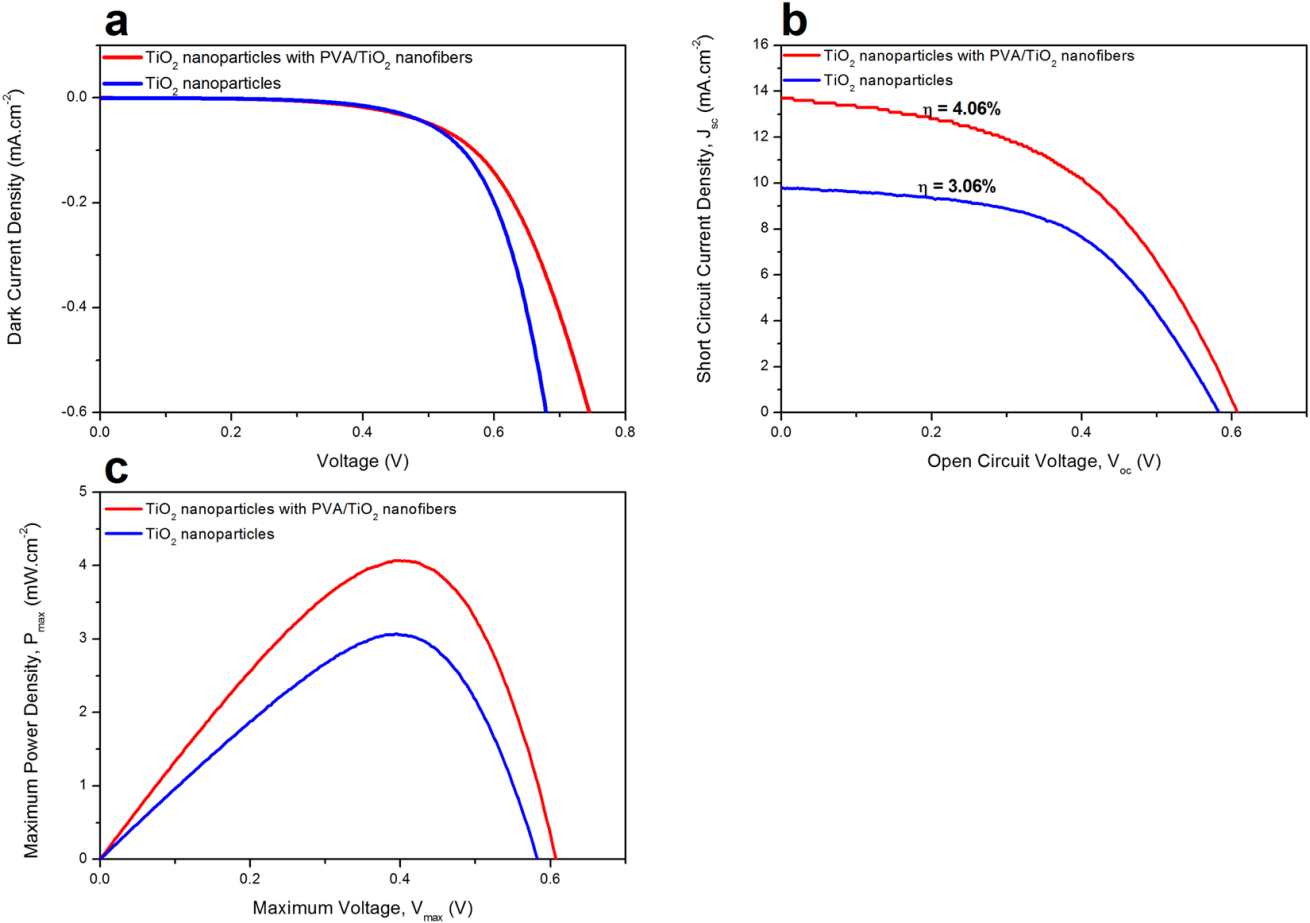
(**a**) Dark J-V curve, (**b**) J-V curve under illumination and (**c**) P-V curve of TiO_2_ nanoparticles and TiO_2_ nanoparticles with PVA/TiO_2_ nanofibers.

Dark current-voltage (J-V) curve analysis was performed to investigate the back electron transfer process in DSSC. Figure 7a shows the dark J-V curve of TiO_2_ nanoparticles and TiO_2_ nanoparticles with PVA/TiO_2_ nanofibers. The onset of the dark currents for TiO_2_ nanoparticles and TiO_2_ nanoparticles with PVA/TiO_2_ nanofibers occur at around 0.58 and 0.62 V, indicating reduce in back electron transfer for a latter compared to a former photoanode. J-V curves were performed under 1 sun illumination with A.M 1.5 G to investigate the total PCE generated by TiO_2_ nanoparticles and TiO_2_ nanoparticles with PVA/TiO_2_ nanofibers. As shown in Fig. 7b, the TiO_2_ nanoparticles with PVA/TiO_2_ nanofibers display a higher PCE of 4.09% compared to the TiO_2_ nanoparticles (3.06%). Figure 7c shows that upon addition of PVA/TiO_2_ nanofibers as LSL on the photoanode, the maximum power generated by the DSSC increases. Table 2 summarizes and compares the photovoltaic performance of the DSSC devices. The photoanode with LSL shows a vast increment of PCE which about 33% more than the photoanode without LSL, indicating the incorporation of PVA/TiO_2_ nanofibers as LSL improves the current and voltage generated throughout the DSSC process that leads to high PCE produces. This fact is supported by the increment of both short circuit current density (*J*_sc_) and open circuit voltage (*V*_oc_) of photoanode with PVA/TiO_2_ nanofibers as LSL compared to the photoanode without LSL. The increment of both *J*_sc_ and *V*_oc_ after incorporation of LSL demonstrates that the LSL help to reduce the radiation loss during DSSC process^27,28^. This extra radiation helps to excite more electrons of the dye molecules, producing more electron-hole junction that leads to more power to be generated. As a consequence, the maximum voltage and maximum power of the DSSC device with PVA/TiO_2_ nanofibers as LSL increases. Even though the total PCE of the DSSC device with PVA/TiO_2_ nanofibers as LSL improves compared with the DSSC device without LSL, the fill factor undergo a slight drop from 53.89 to 48.58% due to the addition of LSL increases the internal resistant of photoanode^28^. This result is in agreement with the EIS analysis where an increase in *R*_ct_ is observed upon addition of PVA/TiO_2_ nanofibers as LSL. The PCE obtained in this study is comparable with the cadmium doped TiO_2_ nanofibers (2.95%)^22^, sulfur doped TiO_2_ nanofibers (4.27%)^37^ and gold doped TiO_2_ nanofibers (5.08%)^21^.

**Table 2:** Comparison of the Photovoltaic performances of the DSSCs.

Photoanodes	*J*_sc_ (mA/cm²)	*V*_oc_ (V)	*P*_max_ (mW/cm²)	FF (%)	η (%)	References
Cadmium doped TiO_2_ nanofibers	8.73	0.68	2.73	46.20	2.95	^22^
Sulfur doped TiO_2_ nanofibers	10.66	0.68	4.27	59.00	4.27	^37^
Gold doped TiO_2_ nanofibers	10.07	0.76	5.08	76.00	5.08	^21^
TiO_2_ nanoparticles with PVA/TiO_2_ nanofibers	13.70	0.61	4.06	48.58	4.06	This work

## Conclusion

TiO_2_ nanoparticles decorated with PVA/TiO_2_ nanofibers as LSL with remarkably enhanced DSSC performance were successfully synthesized. The PVA/TiO_2_ nanofibers were prepared by facile electrospinning using PVA as a polymer source and TTIP as conductive metal oxide precursor. Upon addition of the PVA/TiO_2_ nanofibers as LSL on top of the photoanode, the PCE of the DSSC device increased 33% compared to the photoanode without LSL. This outstanding enhancement of PCE was attributed to the fact that the LSL reduced the radiation loss, producing more oxidation of dye molecule, resulting in more electrons to be excited and more PCE to be generated.
